# Black Garlic and Thiosulfinate-Enriched Extracts as Adjuvants to Ceftriaxone Treatment in a Rat Peritonitis Model of Sepsis

**DOI:** 10.3390/biomedicines10123095

**Published:** 2022-12-01

**Authors:** Francisco Javier Redondo-Calvo, Natalia Bejarano-Ramírez, Víctor Baladrón, Omar Montenegro, Luis Antonio Gómez, Rubén Velasco, Natalia Villasanti, Soledad Illescas, María Teresa Franco-Sereno, Ignacio Gracia, Juan Francisco Rodríguez, José Ramón Muñoz-Rodríguez, José Manuel Pérez-Ortiz

**Affiliations:** 1 Department of Anesthesiology and Critical Care Medicine, University General Hospital, 13004 Ciudad Real, Spain; 2 Translational Research Unit, University General Hospital and Research Institute of Castilla-La Mancha (IDISCAM), 13004 Ciudad Real, Spain; 3 Faculty of Medicine, Universidad de Castilla-La Mancha, 13071 Ciudad Real, Spain; 4 Department of Pediatrics, University General Hospital, 13004 Ciudad Real, Spain; 5 Department of Chemical Engineering, Institute of Chemical and Environmental Technology, Universidad de Castilla-La Mancha, 13071 Ciudad Real, Spain; 6 Department of Pathological Anatomy, University General Hospital, 13004 Ciudad Real, Spain; 7 Department of Microbiology, University General Hospital, 13004 Ciudad Real, Spain; 8 Department of Pharmacy, University General Hospital, 13004 Ciudad Real, Spain

**Keywords:** garlic, *Allium sativum*, thiosulfinate, allicin, sepsis, immunomodulation, interleukins, rats

## Abstract

To date, there have been no new drugs or adjuvants able to decrease both morbidity and mortality in the context of sepsis and septic shock. Our objective was to evaluate the use of thiosulfinate-enriched *Allium sativum* and black garlic extracts as adjuvants in the management of sepsis. An experimental in vivo study was carried out with male Sprague–Dawley^®^ rats. Animals were randomized in four treatment groups: antibiotic (ceftriaxone) treatment (group I), ceftriaxone plus thiosulfinate-enriched extract (TASE, group II), ceftriaxone plus thiosulfinate-enriched extract and black garlic extracts (TASE + BGE, group III), and ceftriaxone plus black garlic extract (BGE, group IV). All animals were housed and inoculated with 1 × 10^10^ CFU/15 mL of intraperitoneal *Escherichia coli* ATCC 25922. Subsequently, they received a daily treatment according to each group for 7 days. Clinical, analytical, microbiological, and histopathological parameters were evaluated. Statistically significant clinical improvement was observed in rats receiving garlic extracts in weight (groups II and III), ocular secretions, and piloerection (group IV). Moreover, less liver edema, vacuolization, and inflammation were observed in groups receiving adjuvant support (groups II, III, and IV). When comparing interleukins 24 h after bacteria inoculum, we found statistically significant differences in TNF-alpha levels in groups receiving BGE (groups III and IV, *p* ≤ 0.05). Blood and peritoneal liquid cultures were also analyzed, and we detected a certain level of *Enterococcus faecalis* in peritoneal cultures from all treatment groups and less bacteria presence in blood cultures in rats receiving garlic extracts (groups II, III, and IV). In conclusion, TASE and BGE could be promising nutraceutical or medicinal agents as coadjuvants in the treatment of sepsis because of its effects in modulating the inflammatory response.

## 1. Introduction

Sepsis and septic shock, according to the latest international consensus, is a life-threatening organ dysfunction due to an altered host response to infection. In septic shock, we find circulatory and cellular/metabolic alterations that cause organ failure and can increase mortality and be one of the leading causes of death in intensive care units [1,2]. Scales such as the SOFA and qSOFA have been used for monitoring and assessing the severity of organ dysfunction [3], although in the latest Surviving Sepsis Campaign guidelines for management of sepsis and septic shock 2021 the recommendation has been withdrawn due to its low sensitivity for predicting patient mortality [4].

Sepsis initiates with a first inflammatory phase, sometimes exaggerated with the release of different inflammatory mediators that can lead to organ dysfunction (activation of monocytes, macrophages, neutrophils, endothelial cells, and platelets), local and systemic release of cytokines, stimulation of the complement cascade, activation of the coagulation and fibrinolytic system, activation of the nitric oxide pathway, production of free radicals, and stimulation of B and T lymphocytes and their products [5,6]. This can be followed, sometimes even coincidentally and overlapping, by another phase of immunosuppression with a compensatory anti-inflammatory response where we can find endotoxin tolerance, lymphocyte apoptosis, increased regulatory T cells, and changes in the phenotype of monocytes/macrophages that can lead to the appearance of nosocomial/opportunistic infections and viral reactivations [7,8,9].

Thiosulfinates contained in garlic (*Allium sativum*) have been used as antineoplastic and antimicrobial compounds [10,11]. It has been described that allicin could interfere at several points in the phases that occur in sepsis and septic shock: inhibiting the activation of nuclear factor NF-kB [12], preventing the adhesion of T cells to endothelial cells [12], reducing vasodilatation by inhibiting nitric oxide synthase [13], and acting as an antiplatelet [14].

Black garlic extract (BGE) is an odorless product, with a sweet and sour taste and a gelatinous texture that is obtained by the fermentation process of natural garlic at high temperatures (Maillard reaction) and humidity for 3 or 4 weeks. In this process, alliinase, an enzyme capable of hydrolyzing cysteine derivatives that give rise to thiosulfinates, is denatured [15,16]. Of these, alliin is the precursor of allicin and is found in stable concentrations in black garlic, even at room temperature and in aqueous solution. The final product contains high levels of bioactive organic sulfur compounds such as water-soluble S-allyl-L-cysteine, flavonoids, thiosulfates, and polyphenols with antioxidant capacities [17,18,19]. Furthermore, BGE is also able to modulate the activity of NK cells, nitric oxide, interferon-gamma, IL-2 and TNF-alpha [20].

Due to the lack of studies on the anti-inflammatory activity of these combined extracts and the possible interaction in some of the phases that occur in sepsis and septic shock, we have decided to explore whether intraperitoneal applications of TASE, BGE, and a combination could be an adjuvant to specific antibiotic treatment in this clinical syndrome of organ dysfunction caused by a dysregulated response to infection and to evaluate their possible immunomodulatory role.

## 2. Materials and Methods

### 2.1. Animals and Sepsis Model

Male Sprague–Dawley^®^ rats (Harlan Laboratories Models, Barcelona, Spain), 5-week-old, were used. The study was conducted at the Translational Research Unit of the University General Hospital, Ciudad Real. The procedures were carried out at the same time of day to avoid the possible influence of the circadian cycle on the results of the work.

Rats were kept with food and water ad libitum, in a cycle of 12 h of light and 12 h of darkness, and a room temperature of 22 ± 2 °C with a relative humidity of 50–70% and 15–20 air renewals per hour without recirculation. They were housed according to RD 53/2013 and no rat was caged alone to favor their group behavior. In addition, they were maintained in these environmental conditions to allow acclimatization for six days before the study started. Animals were randomized in 4 treatment groups: group I: ceftriaxone (CEF, *n* = 5); group II: ceftriaxone plus thiosulfinate-enriched *Allium sativum* extract (CEF + TASE, *n* = 5); group III: ceftriaxone plus thiosulfinate-enriched and black garlic extracts (CEF + TASE + BGE, *n* = 5); and group IV: ceftriaxone plus black garlic extract (CEF + BGE, *n* = 5). A model of peritonitis was generated in all groups. Rats from different groups were never housed in the same cage.

In a previous study carried outby our group, we established the protocol to generate a sepsis and septic shock model [21]. Specifically, an inoculum of 1 × 10^10^ colony forming units of *Escherichia coli* ATCC 25922 in 15 mL of distilled water was set as optimal and administered intraperitoneally to each anesthetized rat.

### 2.2. Thiosulfinate-Enriched and Black Garlic Extracts

Fresh garlic was obtained from the purple garlic ecotype from Las Pedroñeras, the only European region with Protected Geographical Status for garlic (ES/PGI/005/0228/12.03.2002), and generously supplied by “Ajos El Molino” (Las Pedroñeras, Cuenca, Spain). Black garlic was obtained from the same purple garlic ecotype and used to produce freeze-dried black garlic extract with standardized S-allyl-L-cysteine (SAC) content following a previous protocol [20]. Specifically, black garlic extract was created from fresh purple garlic by processing (aging) it in a temperature (65–80 °C) and humidity (70–80%)-controlled room for a month.

Solvents used were ammonium formate (purity 99.5%), methanol (Isocratic-preparative HPLC), ethanol (purity 96% *v*/*v*), deionized water (Milli-Q standard), and physiological serum (NaCl 0.9%). For instrumental analysis, SAC (purity 99%) and dimethyl sulfoxide (purity 99%) were employed as internal and external standards, respectively. Reagents were obtained from Panreac Quimica (Barcelona, Spain).

For the obtention of a thiosulfinate-enriched *Allium sativum* extract (TASE), the raw material was subjected to extraction and HPLC analysis according to a previous report [22]. For this study, we obtained a standardized, formulable, and soluble allicin preparation from fresh garlic of 7.03 mg/g, as shown in Table 1.

For the obtention of the standardized SAC soluble material, black garlic was processed as follows: black garlic was milled and cut using a Thermomix 31-1 model mincer (Vorwerk, Madrid, Spain) to give fractions with an average diameter between 1–3 mm. Milled black garlic was homogenated with physiological serum (NaCl 0.9%) in a 2 L stirred-tank extractor to maximize the yield of SAC. Once obtaining a homogeneous mixture in the form of a paste, the mixture was subjected to extraction at 50 °C for 120 min with a marine propeller agitator. The stirring system consisted of a jar-test Vittadini 6-P model (Isco, Rome, Italy) with digital control of the stirring speed, which was set at 145 rpm. After extraction, the extract was subjected to centrifugation in 50 mL vials at 4000 rpm for 15 min. The supernatant was fully and carefully collected by pipetting, and the solid phase remained as a residue at the bottom of the vials. The extract was then filtered through 15 mm × 0.45 µm plastic filters under positive pressure. The extract was freeze-dried as previously reported [23] to obtain a standardized, formulable and soluble SAC lyophilized preparation from BGE with a particular concentration in this study of 0.31 mg/g, as shown in Table 1.

For biological tests, standardized SAC or allicin were dissolved in physiological serum until appropriate concentration. The liquid solution obtained was kept cold at 4 °C until use and formulated by the Department of Pharmacy. A 3 mL aliquot was destined to chromatographic analysis of allicin or SAC [21], according to the established HPLC conditions. The stability of allicin and SAC were tested monthly for 1 year and their concentrations were invariable over time as expected in the lyophilized BGE and TASE obtained. Neither allicin nor other thiosulfinates were found in BGE.

### 2.3. Experimental Design and Analytical Parameters

**Figure 1:**
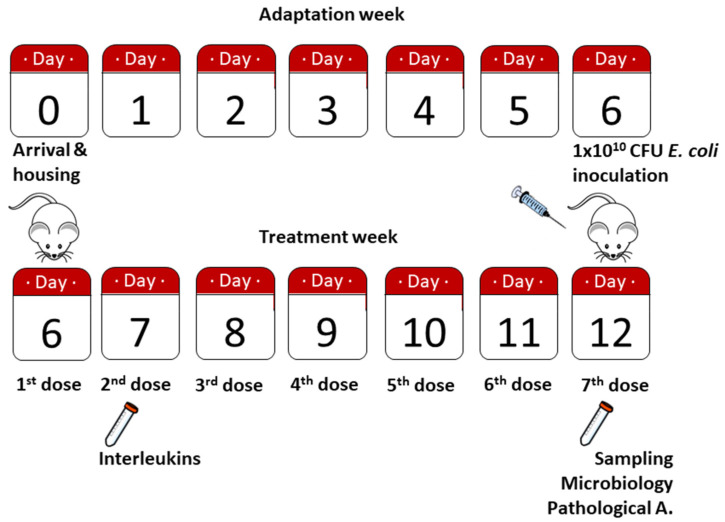
Timeline of the study. Animals were housed and identified on day 0. After 6 days of adaptation to the animal facility, *E. coli* inoculation and first treatment dose were administered; 24 h after, blood samples for interleukin determination were obtained. On day 12, animals were sacrificed by lethal doses of anesthesia, sampled for microbiological analysis (blood and peritoneal fluid) and pathological anatomy (liver and peritoneum).

According to the treatments employed, rats from group I were inoculated with 4.4 mL of antibiotic ceftriaxone (100 mg/kg), rats from group II received 4.4 mL of ceftriaxone (100 mg/kg) + TASE (0.5 mg/kg; referred to allicin content), rats from group III received 4.4 mL of ceftriaxone (100 mg/kg) + TASE (0.5 mg/kg) + BGE (50 µg/kg; referred to S-allyl-L-cysteine content), and group IV the same volume with ceftriaxone (100 mg/kg) + BGE (50 µg/kg). In Figure 1, we show the experimental scheme of the study.

Physical and clinical parameters were evaluated daily by the same person: weight, appearance, ocular secretions, nasal secretions, whisker position, piloerection, level of hydration, abdominal distension, hardening distension, and behavior (normal, hypoactive, and lethargy).

Interleukin (IL) 1β/IL-1F2, IL-6, and Tumor Necrosis Factor alpha (TNF-α) were determined in blood samples on treatment day 2 (T2) with the corresponding Quantikine^®^ Rat ELISA method (R&D Systems) following the manufacturer’s instructions. All samples were diluted 1:3 in RD5Y diluent. Each diluted sample and standards were processed in duplicate. Internal quality control was performed with recombinant buffered IL control material of known concentration. The final reading was made at 450 nm and corrected to 550 nm in a microplate reader.

Peritoneal fluid was also sampled on T7 (last day of the experiment) directly from the peritoneal cavity. A peritoneal fluid study was performed to determine cellularity by Giemsa staining. A blinded expert microbiologist evaluated the samples. During the exploratory laparotomy, the degree of peritoneal inflammation was evaluated macroscopically. Liver and peritoneum samples were obtained for histopathological evaluation. Thus, samples were paraformaldehyde fixed, paraffin embedded, and 4 µm sections were made for hematoxylin/eosin staining to analyze the presence or absence of congestion and immune cells. A blinded expert pathologist evaluated the samples.

### 2.4. Statistical Analysis

A descriptive statistic of the quantitative variables was carried out to verify that the minimum and maximum values were in an adequate range. All data were expressed as mean ± standard error of the mean (x ± SEM).

To analyze the qualitative variables, proportions were compared with the Chi-Square test and Fisher’s exact correlation. The means of the continuous variables were compared with U Mann–Whitney’s test and normal distribution was verified by Shapiro–Wilk’s test. In comparisons at different times within the same groups, tests were applied for paired variables (Student’s *t*-test for dependent variables or Wilcoxon test as the case may be). For the comparison between groups, the Kruskal–Wallis test was used as a function of normality. A significance level of 95% was used for statistical analysis. SPSS 28.0 for Windows (SPSS Inc., Chicago, IL, USA).

## 3. Results

Our model of abdominal sepsis elicits 100% lethality in rats if no antibiotic treatment is provided after 4–6 h post-inoculum [21]. Thus, each group of rats received the first dose of its corresponding treatment on day 6 (*E. coli* inoculation day), and every other day until day 12 (end of the experiment) (Figure 1).

**Figure 2:**
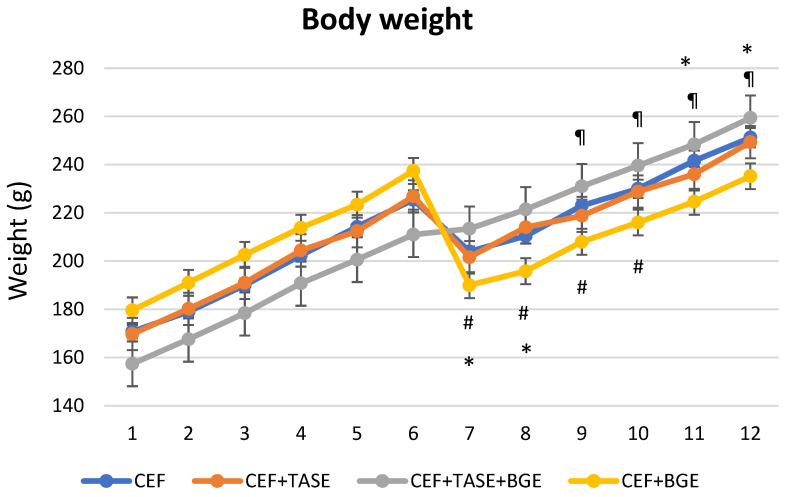
Weight monitoring during experiment (12 days). On day 6, the bacterial inoculum was introduced. CEF = ceftriaxone (* *p* < 0.05). CEF + TASE = ceftriaxone + thiosulfinate-enriched *Allium sativum* extract. CEF + TASE + BGE = ceftriaxone + thiosulfinate-enriched extract + black garlic extract (^¶^ *p* < 0.05). CEF + BGE = ceftriaxone + black garlic extract (# *p* < 0.05). Mean ± SEM.

In relation to clinical parameters, we only found no differences when comparing body weight between day 6 and the following days in CEF + TASE group (Figure 2). For the rest of the groups, CEF treated rats significantly decreased in weight on day 7 and 8, and were able to gain weight on days 11 and 12. CEF + TASE + BGE treated rats not only did not decrease body weight on day 7 and 8, but increased in average weight on days 9, 10, 11, and 12. In the CEF + BGE group, we detected a significant drop in body weight on days 7, 8, 9, and 10 (Figure 2).

In reference to stress and suffering (nasal secretions, ocular secretions, whiskers position, piloerection, and mobility), we could only find differences in the level of ocular secretions 48 h post-inoculum (T2) in the CEF + TASE group with respect to the other treatment groups. Statistically significant differences were also observed in the piloerection of CEF + BGE group, where those rats showed a better hair state at T2 and T3. No statistically significant differences were found in the rest of the clinical signs studied, and at T4 and T5 (Table 2).

**Figure 3:**
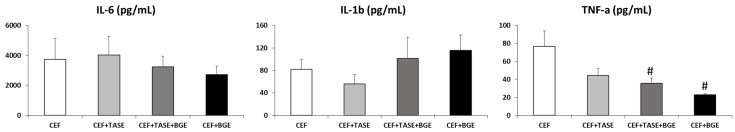
Interleukin levels in T2 (treatment day 2). CEF = ceftriaxone. CEF + TASE = ceftriaxone + thiosulfinate-enriched *Allium sativum* extract. CEF + TASE + BGE = ceftriaxone + thiosulfinate-enriched extract + black garlic extract. CEF + BGE = ceftriaxone + black garlic extract. Mean ± SEM. # *p* < 0.05.

In relation to the biochemical parameters studied (IL-1, IL-6, and TNF-α), a comparison was made for the values of each interleukin between treated groups in T2 (Figure 3). Considering the levels of IL-1 and IL-6, no differences between groups were assessed. As for TNF-α, we found statistically significant differences when comparing group CEF + TASE + BGE and group CEF + BGE with respect to group CEF and group CEF + TASE, as they both showed lower levels of TNF-α expression (*p* < 0.05).

The microbiological analysis of peritoneal liquid cultures showed that *Enterococcus faecalis* was present at a certain level in all treatment groups, although in one rat from the CEF + BGE group, it was undetected (Table 3). Regarding the analysis of blood cultures, two out five rats in the CEF group were negative for bacteria presence; four out five rats in the CEF + TASE group were negative for bacteria presence; four out five rats in the CEF + TASE + BGE group were also negative for bacteria presence; and three out five rats in CEF + BGE group were negative for bacteria presence.

Regarding the histopathological analysis and organ evaluation, the inflammatory cell count, as well as the congestion and hepatic vacuolization between treatment groups, statistically significant differences were found in some parameters (Table 4). The CEF + TASE + BGE group did not show any evident sign of hepatic congestion or leukocytes presence. Likewise, any rat from the CEF + BGE group displayed PMN leukocytes in the liver. Remarkably, all the groups that included TASE, BGE, or both as adjuvants to antibiotic treatment showed no indication of hepatic vacuolization and a better edema state.

## 4. Discussion

Despite the continuous effort and research [24,25,26,27], the pathophysiology of sepsis is still not fully elucidated. In most occasions, sepsis shows a dynamic nature, with important differences in the innate response of the different animal models employed [28,29]. Consequently, there is a lack of robust biomarkers [30] and few interventions and approaches to treatment that have shown to be beneficial other than antimicrobial therapy and fluid resuscitation [31].

SAC and allicin concentrations were used as the reference for BGE and TASE experimental treatments by their previous demonstrated bioactivity [22,23]. The concentration of other thiosulfinates were not determined since allicin is the most unstable compound of all thiosulfinates contained in TASE. Thus, a direct correlation between allicin concentration and biological activity has been previously established. Although synergistic effects have been detected between components, the presence of allicin is a determining factor [32].

Proinflammatory cytokines, such as TNF-alpha, IL-1b, and IL-6, have diverse effects in the regulation of immune reactions and inflammation [33,34], secondary to sepsis, hence they were the ones determined in our study. Specifically, TNF-alpha is one of the best studied cytokines in sepsis, being able to stimulate the expression of a wide variety of genes involving the myocardium and the surrounding tissue environment [35].

Looking for the immunomodulatory capacity of compounds derived from garlic, this study has been designed using two extracts (TASE and BGE) and its combination with antibiotic, finding a decrease in TNF-alpha in the first 24 h in the two groups where BGE was included. In a previous work [23], we compared only TASE to antibiotic treatment in T3 and T7, and we did not find a significant decrease in TNF-alpha. In the present work, we also found no differences in any of the cytokines analyzed at T5 (i.e., 120 h later), which falls within the context of regulation of the host inflammatory response, where toxins are eliminated or neutralized 24 h after contact [36].

An in vitro study showed that BGE significantly suppressed nitric oxide (NO) and TNF-alpha production in a dose-dependent manner in lipopolysaccharide (LPS)-stimulated RAW264.7 macrophages [37]. Jeong et al. [38] showed that increased sugar concentrations and decreased allicin concentrations during BGE processing lead to lower anti-inflammatory effects in LPS-activated RAW264.7 cells than fresh garlic. In our study, we have also found a significant decrease in TNF-alpha values in BGE groups. This has also been described by Kim et al., where BGE was able to decrease the inflammatory response by modulating the transcriptional level of TNF-alpha mRNA, IL-6, and iNOS induced by LPS [37].

The polyphenols present in these extracts have been shown to modulate the activity of two redox-sensitive transcription factors. Nuclear factor (NF)-κB was inhibited and NF-E2-related factor was activated, and this appeared to be important for the attenuation of the inflammatory response in sepsis [39]. In lung tissue of LPS-affected rats, allicin exerted its effects through inhibition of the TLR4/MyD88/NF-kB signaling pathway, in addition to inhibiting caspase-3 and caspase-9 activity [40].

It has also been shown that BGE could decrease oxidative stress by inhibiting NF-kB activation in immune cells [41], may be involved in modulating NO production, and may be related to a direct inhibition of inflammation-related transcription factors [42]. However, the cellular mechanisms related to inflammation are not fully understood. The main compounds showing anti-inflammatory effects in BGE are pyruvate, 2-linoleoylglycerol, and 5-hydroxymethylfurfural [38,43,44]. Bayraktar et al. demonstrated that SAC attenuated NO activity in lung tissue and decreased apoptosis in hepatocytes [45]. In addition, BGE can achieve the decrease of sepsis-induced lung inflammation by reducing the expression of iNOS and COX-2 [46].

Regarding weight status, rats infused with TASE not only did not show a significant drop in body weight but their body mass increased more quickly in the TASE + BGE group, highlighting that TASE can reduce the impact of an infection/inoculum, as previously described [23].

All this also correlates with other clinical and anatomopathological signs considered in this work. Few studies with animal models associate analysis of histological findings in intraperitoneal organs such as liver and peritoneum after peritonitis and treatment with garlic extracts [23]. Only Lee et al. [47] have examined inflammatory changes in lungs. Thus, we observed that groups that incorporate BGE in their treatment exhibited significantly fewer ocular secretions and less piloerection on T2 and T3. Those same groups displayed significantly fewer sinusoidal PMN leukocytes and perinuclear vacuolization, and no sign of hepatic congestion was detected with extracts combinations (CEF + TASE + BGE).

The microbiological analysis showed that none of the blood cultures from the different groups have the strain of *E. coli* used as inoculum for intraperitoneal infection, demonstrating the effectiveness of the treatments used. It should be noted that a small proportion of the blood cultures and 19 out of 20 peritoneal fluid cultures showed the presence of *Enterococcus faecalis*, due to the selection of these strains because of the change that gastrointestinal microbiota undergoes after treatment with an antibiotic such as broad-spectrum ceftriaxone. We know that Gram-negative bacteria stimulate the production of RegIII (Lecithin Receptor type C) and thus maintain the balance between the bacteria that make up the intestinal microbiota and the host [48]. The use of ceftriaxone would lead to a decrease in Gram-negative bacteria and in the production of RegIII and with it the overgrowth of Gram-positive cocci (*Enterococcus faecalis*), which can cross the intestinal barrier and reach the bloodstream. It is worth noting that the groups where the adjuvants were present (TASE, BGE, or both) showed the fewest positive blood cultures.

Our study has several limitations. First, goal-directed therapy was not possible as it is usually carried out in humans with sepsis, for which continuous macrodynamics measurements (i.e., cardiac output, preload, vascular resistance) and microhemodynamics measurements are required. Second, the blood obtained was limited and did not allow us to determine more proinflammatory factors and markers of tissue hypoxia and ischemia/reperfusion.

In addition, the components of BGE are not yet fully described and the components and their concentrations may vary with aging time and temperature. Moreover, the variation in physicochemical properties and concentrations of the components could be influenced by the cultivation areas and the different varieties used [49,50]. Therefore, further studies are needed to understand in more detail the biological functions and composition of BGE.

## 5. Conclusions

We can conclude that BGE or the mixture with TASE could be a promising nutraceutical or medicinal agent as coadjuvant in the treatment of sepsis because of its effects in modulating the inflammatory response. In fact, improvements detected on physical parameters, inflammation level, bacteria presence, and decrease on TNF-alpha in animals treated with TASE and BGE make those extracts worth further exploring.

## 6. Patents

Patent WO 2008/102036 A1. Method for obtaining a freeze-dried, stable extract from plants of the *Allium* genus.

National patent (Spanish Trademark number ES2675282A1). *Allium sativum* extract, its use for the manufacture of a medicinal product for the treatment of diseases, and its obtaining procedure.

## Figures and Tables

**Table 1:** Composition of organic compounds of lyophilized thiosulfinate-enriched *Allium sativum* extract (TASE) and black garlic extract (BGE) from purple garlic of Las Pedroñeras (Cuenca, Spain) used in this study.

Compound	TASE (mg/g)	BGE (mg/g)
Total polyphenols (mg GAE/g) *	13.91	48.35
Total flavonoids (mg RE/g) **	3.22	5.38
Diallyl thiosulfinate (allicin)	7.03	ND
S-allyl-L-cysteine	0.08	0.31
Leucine	0.586	0.592
Isoleucine	0.500	0.711
Valine	0.477	0.339
Methionine	0.316	0.711
Cysteine	0.811	0.421
Phenylalanine	0.556	1.431
Tyrosine	4.499	0.773
Aspartic Acid	0.901	0.602
Glutamic Acid	2.866	1.001
Arginine	4.090	0.403
Lysine	0.617	0.405
Histidine	0.891	0.579
Threonine	0.812	0.594
Serine	0.385	0.237
Glycine	0.215	ND
Alanine	0.897	0.327
Thiamine (B1)	0.552	ND
Riboflavin (B2)	0.002	ND
Niacin (B3)	0.026	0.163
Pantothenic acid (B5)	1.556	9.927
Biotin (B7)	0.251	0.040
Cobalamin (B12)	0.898	ND
Ascorbic Acid (C)	3.347	2.613
Linoleic Acid (F)	0.276	0.243
Tocopherol (E)	0.007	0.0001
Menadione (K3)	0.007	0.005

ND = non-detected; * mg gallic acid equivalents/g; ** mg rutin equivalents/g.

**Table 2:** Clinical parameters in relation to stress and suffering at T1, T2, T3, T4, and T5 (24, 48, 72, 96, and 120 h post-inoculum, respectively) for nasal secretions, eye secretions, position of whiskers, hair erection, and hypoactivity. * *p* ≤ 0.05.

	T1				
Variable, n/nt	CEF	CEF + TASE	CEF + TASE + BGE	CEF + BGE	*p*-Value
Nasal secretions 1	0/5	2/5	1/5	2/5	0.402
Ocular secretions 1	2/5	2/5	2/5	4/5	0.115
Whiskers separation 1	3/5	3/5	5/5	4/5	0.402
Piloerection 1	5/5	5/5	5/5	5/5	-
Hypoactivity 1	0/5	1/5	2/5	1/5	0.475
	**T2**				
**Variable, n/nt**	**CEF**	**CEF + TASE**	**CEF + TASE + BGE**	**CEF + BGE**	***p*-Value**
Nasal secretions 2	3/5	1/5	0/5	1/5	0.167
Ocular secretions 2	1/5	4/5	0/5	1/5	0.036 *
Whiskers separation 2	4/5	5/5	5/5	4/5	0.528
Piloerection 2	5/5	5/5	5/5	2/5	0.014 *
Hypoactivity 2	0/5	0/5	0/5	0/5	-
	**T3**				
**Variable, n/nt**	**CEF**	**CEF + TASE**	**CEF + TASE + BGE**	**CEF + BGE**	***p*-Value**
Nasal secretions 3	2/5	2/5	1/5	1/5	0.813
Ocular secretions 3	0/5	0/5	0/5	0/5	-
Whiskers separation 3	5/5	5/5	5/5	5/5	-
Piloerection 3	3/5	5/5	4/5	0/5	0.009 *
Hypoactivity 3	0/5	0/5	0/5	1/5	0.368
	**T4**				
**Variable, n/nt**	**CEF**	**CEF + TASE**	**CEF + TASE + BGE**	**CEF + BGE**	***p*-Value**
Nasal secretions 4	2/5	0/5	0/5	1/5	0.230
Ocular secretions 4	0/5	0/5	0/5	0/5	-
Whiskers separation 4	5/5	5/5	5/5	5/5	-
Piloerection 4	1/5	1/5	0/5	0/5	0.528
Hypoactivity 4	0/5	0/5	0/5	0/5	-
	**T5**				
**Variable, n/nt**	**CEF**	**CEF + TASE**	**CEF + TASE + BGE**	**CEF + BGE**	***p*-Value**
Nasal secretions 5	0/5	0/5	0/5	0/5	-
Ocular secretions 5	0/5	0/5	0/5	0/5	-
Whiskers separation 5	5/5	5/5	5/5	5/5	-
Piloerection 5	1/5	0/5	0/5	0/5	0.368
Hypoactivity 5	0/5	0/5	1/5	0/5	0.368

**Table 3:** Results of blood and peritoneal fluid cultures stratified by treatment groups.

Results of Microbiological Studies		
	Peritoneal Liquid Cultures (*E. faecalis*)	Blood Cultures
Ceftriaxone (Group I, *n* = 5)	1400 (1)	*Enterococcus faecalis* (2)
	≤100 (4)	*Staphylococcus sciuri* (1)
		Negative (2)
Ceftriaxone + TASE (Group II, *n* = 5)		*Enterococcus faecalis* (1)
	≤100 (5)	Negative (4)
Ceftriaxone + TASE + BGE (Group III, *n* = 5)		*Enterococcus faecalis* (1)
	≤100 (5)	Negative (4)
Ceftriaxone + BGE (Group IV, *n* = 5)	200 (1)	*Enterococcus faecalis* (1)
	≤100 (3)	*Staphylococcus capitis* (1)
	Non-detected (1)	Negative (3)

**Table 4:** Histopathological analysis and organ evaluation in relation to inflammation, congestion, and vacuolization. * *p* ≤ 0.05.

Variable, n/nt	CEF	CEF + TASE	CEF + TASE + BGE	CEF + BGE	*p*-Value
Liver—hepatic congestion	4/5	5/5	0/5	4/5	0.005 *
Liver—sinusoidal PMN leukocytes	2/5	4/5	0/5	0/5	0.015 *
Liver—edema	5/5	3/5	2/5	1/5	0.070
Liver—necrosis	0/5	2/5	0/5	0/5	0.083
Liver—perinuclear vacuolization	3/5	0/5	0/5	0/5	0.014 *
Peritoneum—inflammation	3/5	5/5	3/5	4/5	0.402

## Data Availability

Not applicable.
